# The Employment of Leukotriene Antagonists in Cutaneous Diseases Belonging to Allergological Field

**DOI:** 10.1155/2010/628171

**Published:** 2010-09-22

**Authors:** Eustachio Nettis, Maddalena D'Erasmo, Elisabetta Di Leo, Gianfranco Calogiuri, Vincenzo Montinaro, Antonio Ferrannini, Angelo Vacca

**Affiliations:** ^1^Section of Allergology and Clinical Immunology, Department of Internal Medicine and Infectious Diseases, University of Bari Medical School, Piazza Giulio Cesare, 70124 Bari, Italy; ^2^Fourth Pneumology Department, Pneumologic Hospital A. Galateo, San Cesario di Lecce, Italy; ^3^Division of Nephrology, Department of Emergency and Organ Transplantation, University of Bari, Azienda Ospedaliera “Policlinico”, 70124 Bari, Italy; ^4^Department of Internal Medicine and Clinical Oncology, University of Bari Medical School, 70124 Bari, Italy

## Abstract

Leukotrienes (LTs) are potent biological proinflammatory mediators. LTC4, LTD4, and LTE4 are more frequently involved in chronic inflammatory responses and exert their actions binding to a cysteinyl-LT 1 (CysLT1) receptor and a cysteinyl-LT 2 (CysLT2) receptor. LTs receptor antagonists available for clinical use demonstrate high-affinity binding to the CysLT1 receptor. In this paper the employment of anti-LTs in allergic cutaneous diseases is analyzed showing that several studies have recently reported a beneficial effects of these agents (montelukast and zafirlukast as well as zileuton) for the treatment of some allergic cutaneous related diseases-like chronic urticaria and atopic eczema although their proper application remains to be established.

## 1. Introduction

Several studies have recently reported a beneficial effects of leukotriene (LT) receptor antagonists (montelukast and zafirlukast) as well as zileuton, a 5-lipoxygenase inhibitor, for the treatment of some allergic cutaneous related diseases-like chronic urticaria and atopic eczema [1, 2], although their proper application remains to be established [2]. 

Although histamine is considered the principal mediator of immediate allergic responses, other factors (kinins, prostaglandins and LTs) prolong the inflammatory process in the so-called late phase response of allergic reaction [1] thus causing the poorly responsiveness of symptoms to the treatment with antihistamine agents only [3]. 

 Leukotrienes (LTs) are a class of potent biological pro-inflammatory mediators derived from arachidonic acid through the 5-lipoxygenase pathway divided into two groups according to their chemical structure: those with a sulphur linkage or cysteinyl LTs: LTC4, LTD4, LTE4 are more frequently involved in chronic inflammatory responses and exert their actions through the binding to two types of activating receptors: a cysteinyl-LT 1 (CysLT1) receptor and a cysteinyl-LT 2 (CysLT2) receptor [4, 5].

 Eosinophils, basophils and mast cells are the most important sources of cysteinyl-LTs and epidermal cells are able to transform neutrophil-derived LTA4 into LTB4 and LTC4 [6]. Thus the epidermis can also contribute significantly to LT synthesis *in situ*.

 Surely Cys-LTs play a role in promoting and maintaining the allergic inflammatory response in cutaneous disease as atopic dermatitis and chronic urticaria as well as in asthma through their active effects on chemotaxis, vasodilatation and oedema, LTs are, in fact, potent spasmogenic and chemotactic agents that increase capillary and small vessels permeability. When LTs are injected into human skin, they cause wheal and flare reactions [7], with an action 100-fold more potent than histamine, and consequent sensory nerve stimulation that provokes itching and pain [8, 9]. 

 There is evidence of enhanced LT production in the pathogenesis of AD. Patients with AD have activated circulating basophils and increased basophil and neutrophil releasability of LT-C4 compared with healthy subjects [10, 11], while urinary levels of LT-E4, a stable metabolite of LT-C4 and LT-D4, has been showed high levels in children affected by severe atopic eczema, but not in healthy normal subjects or in patients with mild or moderate atopic eczema [12]. The role of Leukotrienes in allergological cutaneous diseases is shown in Table 1.

 Actually, three LTs receptor antagonists are available for clinical use, montelukast, used in patients older than 6 years, and zafirlukast, approved for adolescent and adult subjects. Montelukast, Zafirlukast and Pranlukast [13] are LT receptor antagonists that demonstrate high-affinity binding to the CysLT1 receptor.

## 2. Leukotriene Receptor Antagonists in Chronic Urticaria

The EEACI/GA²LEN/EDF guideline for the management of urticaria is a consensus reached during panel discussion at the Second International Consensus Meeting on Urticaria, Urticaria 2004 [14], joint initiative of the EAACI Dermatology Section and GA²LEN and they have been updated recently [15]. According to this guideline, management is divided into three basic approaches.

 First approach is avoidance, elimination or treatment of eliciting stimulus or cause. This is the best way since identification of the cause allows successful treatment; however, it may not be possible in all cases. It includes elimination of medicaments, physical stimuli, eradication of infectious agents and treatment of inflammatory processes, and also removal of autoantibodies to the high-affinity IgE receptor. It is applicable in patients with IgE-mediated or physical urticaria. Second approach is inhibition of mast cell mediator release and nowadays the most commonly used drugs inhibiting mast cell release are corticosteroids. Other drugs with inhibiting activity on mast cells are, for example cyclosporin A and PUVA therapy. In the light of these considerations, even the LTs receptor antagonists could play a their role in the treatment of chronic urticaria [1, 16]. 

 The chronic urticaria shows different complex aspects in its pathogenesis: approximately 45% of patients with chronic urticaria have an IgG autoantibody directed to the alphasubunit of the high-affinity IgE receptor leading to cutaneous mast cell and basophil activation [17], but it has been recently evidenced that the coagulation cascade and fibrinolysis activation could be involved in the pathomechanism of chronic urticaria [18], whom contribute to form serum histamine releasing antibodies.

 At last the third approach to urticaria is based on therapy to target organ, for example, antihistamines, according to EEACI/GA²LEN/EDF guideline [15], but chronic urticaria is often difficult to treat and may not be controlled with the conventional antihistamine therapy alone [19], despite the new generation antihistamines such as cetirizine, levocetirizine, loratadine, desloratadine and fexofenadine provide both antiallergic and antiinflammatory effects such as inhibition of cytokines release from basophiles and mast cells as well as reduction of chemotactic activity of eosinophils [20].

 Based on the important role of LTs in the pathophysiologic mechanisms of allergic inflammation, antiLT receptors, montelukast and zafirlukast, have recently been used, either as monotherapy or in combination with H1-receptor antagonists, to treat different forms of urticaria [21]. 

 The results of several studies have indicated a positive therapeutic effect of antiLTs in such different conditions as chronic urticaria, (especially the severe urticaria-angioedema induced by acetylsalicylic acid (ASA) and by other nonsteroidal antiinflammatory drugs), cold urticaria, urticaria related to food additives, chronic autoimmune urticaria, steroid-dependent urticaria, delayed-pressure urticaria, chronic idiopathic urticaria and, finally, dermographism [13, 22–25]. 

 A single study reported that pranlukast may provoke urticaria in patients with ASA-induced urticaria; however, this molecule is not available in Europe [26]. 

A better therapeutic effect of montelukast versus cetirizine or placebo has been demonstrated by Pacor et al. in a double-blind, placebo-controlled trial in patients suffering from chronic urticaria related to food additive and/or ASA intolerance [27]. The same authors also studied 160 patients afflicted by moderate chronic idiopathic urticaria. In this study, patients were divided into four harms: the first harm received montelukast alone, the second harm was treated with montelukast *plus* desloratadine, patients in the third harm were treated with desloratadine and, finally, the fourth harm received desloratadine with placebo. This study showed that the therapeutic regimen based on the association of monteleukast and desloratadine was effective in controlling symptoms of urticaria, even though the second drug proved more efficacious than the LTs antagonist [28]. In light of their observations, the authors supported the efficacy of a combination of antiLTs and nonsedating antihistamine for the treatment of urticaria elicited by a well known factor, such as ASA or food additives-induced urtricaria, autoimmune urticaria, acquired cold urticaria and delayed pressure urticaria. While the association of LT receptor antagonists and H1-antihistamine drugs in patients suffering from idiopathic urticaria, according to the same AA., would not add any beneficial effect, compared with the antihistamine monotherapy [28]. Bagenstose and colleagen. obtained similar results: they observed a beneficial effect from a combined treatment with zafirlukast and cetirizine only in patients affected by severe autoimmune urticaria, showing a positive skin response to autologous serum test [29]. 

 According to Nettis et al. positive and better results in terms of improvement of symptoms were obtained with a treatment based on montelukast alone, compared with fexofenadine in patients suffering from chronic idiopathic urticaria; in the same patients these AA. also demonstrated a reduction of wheal performing the autologous serum test after montelukast treatment [30]. 

In another randomized, double-blind, placebo-controlled study on patients with mild chronic urticaria, Nettis also demonstrated that the concomitant administration of desloratadine and montelukast provides a significant improvement in overall urticaria conditions, compared with placebo and desloratadine alone [31]. Effectiveness of therapy with antiLTs in the treatment of chronic idiopathic urticaria has also been demonstrated by Erbagci [32]. He conducted a single-blind, placebo-controlled, cross-over clinical study with montelukast versus placebo, using nonsedating antihistamine when needed. In this study, he showed that montelukast is an effective and safe therapeutic agent in the treatment of refractory chronic idiopathic urticaria [32]. 

Norris and Sullivan, studying LTs and cytokines in steroid-dependent urticaria, found that 60% of patients enrolled in the study manifested a significant improvement of their severe symptoms taking zafirlukast in combination with antihistamines [33].

Sanada et al. confirmed the effectiveness of montelukast in chronic urticaria unresponsive to the antihistamine treatment and, at variance from other observations, they did not reported differences between patients with positive skin reactions to autologous sera and/or those with ASA hypersensitivity. While critical factors were represented by age and duration of symptoms, whereby young patients having a illness for short duration, were more responsive to the treatment with montelukast [34].

 Asero showed a nearly total remission of the disease in the half of twelve patients with unremitting, steroid-dependent urticaria, after treatment with montelukast 10 mg once a day or zafirlukast 20 mg twice a day. Therefore, according to Asero and on the basis of the safety, tolerability and low cost, LT receptor antagonists should be administered in all patients with steroid-dependent chronic urticaria, unresponsive to other therapies [35]. 

 Reimers et al. by realizing a double-blind, placebo-controlled, cross-over study with 20 mg daily of zafirlukast, demonstrated the ineffectiveness of antiLTs in the treatment of chronic urticaria, concluding that LTs have no significant role in the aetiology of this disease. However, evaluating their results, it is important to consider that they observed in 19 cases (41.3%) a resolution of chronic urticaria that was interpreted as a spontaneous remission, on the basis of the high variability of the course of chronic urticaria [36]. 

Regarding to this aspect, Nettis et al. observed that the remission or improvement of the urticaria induced by antiLTs therapy could not be considered as a spontaneous remission because the excellent results were recorded after 3 weeks of active treatment [30]. Further, Nettis and coll. demonstrated the effectiveness, high tolerability and safety of montelukast also in delayed pressure urticaria [37]. In particular, comparing loratadine *plus* montelukast versus loratadine monotherapy, administered for two weeks, the authors reported a more marked improvement of this form of urticaria in patients treated with montelukast combined with loratadine than loratadine alone. They described a negative result of the rechallenge test in 80% of patients enrolled to the combined therapy versus 20% of subjects that received loratadine monotherapy [37]. In another group of delayed pressure urticaria patients, the same authors tested the treatment with desloratadine in association with montelukast versus desloratadine alone, reporting encouraging results. In fact, they observed that both, desloratadine alone and desloratadine *plus* montelukast, improved urticaria in respect to the baseline assessment. However, the combination of antihistamine with antiLTs resulted more efficacious in the suppression of symptoms and the wheal with dermographometer challenge test [38].

A successful treatment of delayed pressure urticaria with montelukast has been reported also by Berkun and Shalit [39]. He described a case of a patient with severe steroid-dependent delayed pressure urticaria, not-responding to several different antihistamines and other therapies. In this patient, the administration of 10 mg daily of montelukast induced the remission of clinical manifestations after one week of treatment [39].

Finally, since Asero has suggested a common link among chronic urticaria, NSAIDs cutaneous hypersensitivity and alterations of coagulative cascade [40, 41] some single cases have been reported by different authors investigating the promising use of LT receptors inhibitors as prevention care of severe urticaria/angioedema exacerbations following NSAIDs assumption in patients with chronic urticaria [42–44], even if neither clinical nor observational study enrolling large groups of these patients has been ever performed as well as in NSAIDs intolerant asthmatics [45]. Curiously, it has been showed low doses of pranlukast seem to induce urticarial eruption in aspirin sensitive patients as paradox effect [26].

## 3. Leukotriene Receptor Antagonist and Atopic Eczema

LTs are also involved in the pathogenesis of other atopic skin disorders. In particular, atopic dermatitis or Atopic Eczema Dermatitis Syndrome (AEDS), similarly to the different forms of urticaria, are associated with infiltration and activation of mast cells and consequent release of vasoactive and pro-inflammatory mediators at the cutaneous level. This mechanism is indirectly supported by the results of some studies which revealed the effectiveness of LTs receptor antagonists in the treatment of AEDS. In this connection, Nettis and colleagues showed, in a placebo-controlled study, that a 6-week treatment with montelukast was effective in inducing a moderate reduction of cutaneous inflammation, as evaluated by the SCORAD index, in 20 adults suffering from moderate to severe AEDS [46].

 Positive effects of therapy with antiLTs in AEDS have also been reported by other authors. Yanase and David-Bajar showed a statistically significant improvement of the clinical manifestations of atopic dermatitis of moderate severity in adult patients receiving 10 mg of montelukast for 8 weeks [47]. In the study of Pei and colleagen, young AEDS patients were treated with montelukast 5 mg, once a day for 4 weeks. At the end of the study, patients receiving the drug had a beneficial effect, compared to the placebo-treated group [48]. Similarly, Carucci et al. reported efficacy of zafirlukast treatment in the same disease [49]. Angelova-Fisher and Tsankov demonstrated that montelukast, used as single therapeutic agent, was capable to improve significantly the clinical manifestations of severe AEDS. In particular, they described a reduction of erythema and exudation after 10 days of treatment with montelukast monotherapy and an improvement of pruritus just within the first week of treatment, in two AEDS patients [50].

 By contrast, some other interventional studies in atopic eczema were not able to show any superiority of treatments based on LTs antagonist in respect to other treatment options or placebo. In particular, Capello et al. compared two treatment regimens based on (a) 10 mg once a day of montelukast and (b) a combined regimen (orally administered cetirizine and clarythromycin, topical corticosteroids and hydrating preparations), in 32 adult patients with moderate-to-severe atopic dermatitis; no difference in the reduction of the SCORAD index in the two treatment groups was reported [51]. Moreover, Friedmann et al. conducted a randomized, double-blind, placebo-controlled trial which did not demonstrate any efficacy of montelukast over placebo in the treatment of moderately severe adult atopic eczema [52]. These findings were also confirmed by Silverberg and Paller that reported a lack of efficacy administering montelukast or zafirlukast in seven patients with atopic dermatitis. Specifically, the treatment determined only a temporary symptomatic relief [53]. Therefore, in the authors' opinion antiLTs were unable to provide a permanent benefit in patients suffering from diffuse atopic dermatitis [53].

## 4. Conclusion

In conclusion, experimental data suggest that LTs are involved in the allergic inflammation, even though their precise pathogenetic role has not been elucidated [9]. These findings induced several authors to test LTs antagonists in the treatment of chronic urticaria and atopic dermatitis in patients poorly responsive to the conventional therapy. Actually, the available results on the efficacy of antiLTs are encouraging but contrasting and not uniform. Benefits have been reported with antiLTs drugs in some forms of urticaria and atopic dermatitis, although to a different extent, by the several studies. Prospective studies aimed at detecting patients that would benefit from antiLTs drugs are warranted [35]. However, on account of the good tolerability and safety of antiLTs agents, also in long-term therapy [37], a treatment with these drugs should be tried in all cases of urticaria or atopic eczema unresponsive to the conventional therapy.

## Figures and Tables

**Table 1:** Role of Leukotrienes in allergological cutaneous diseases.

Allergological cutaneous diseases	Level and Leukotrienes involved	Cutaneous effects
Chronic Urticaria	Serum increase of Leukotrienes [8]	Chemotactic and spasmogenic effects Sensory nerve stimulation inducing itching and pain [8, 9]
Atopic Dermatitis	Serum increase of Leukotrienes-C4 [10, 11] Urinary levels increase of Leukotrienes -E4 [12]	
